# Livestock-associated *Staphylococcus aureus* on Polish pig farms

**DOI:** 10.1371/journal.pone.0170745

**Published:** 2017-02-02

**Authors:** Aneta Mroczkowska, Jacek Żmudzki, Natalia Marszałek, Monika Orczykowska-Kotyna, Iga Komorowska, Agnieszka Nowak, Anna Grzesiak, Ewelina Czyżewska-Dors, Arkadiusz Dors, Zygmunt Pejsak, Waleria Hryniewicz, Tomasz Wyszomirski, Joanna Empel

**Affiliations:** 1 Department of Epidemiology and Clinical Microbiology, National Medicines Institute, Warsaw, Poland; 2 Department of Swine Diseases, National Veterinary Research Institute, Puławy, Poland; 3 Centre of Quality Control in Microbiology, Warsaw, Poland; 4 Faculty of Biology, Biological and Chemical Research Centre, University of Warsaw, Warsaw, Poland; Universitatsklinikum Munster, GERMANY

## Abstract

**Background:**

Livestock-associated *Staphylococcus aureus* (LA-SA) draws increasing attention due to its particular ability to colonize farm animals and be transmitted to people, which in turn leads to its spread in the environment. The aim of the study was to determine the dissemination of LA-SA on pig farms selected throughout Poland, characterize the population structure of identified *S*. *aureus*, and assess the prevalence of LA-SA carriage amongst farmers and veterinarians being in contact with pigs.

**Methods and findings:**

The study was conducted on 123 pig farms (89 farrow-to-finish and 34 nucleus herds), located in 15 out of 16 provinces of Poland. Human and pig nasal swabs, as well as dust samples were analyzed. *S*. *aureus* was detected on 79 (64.2%) farms from 14 provinces. Amongst these farms LA-SA-positive farms dominated (71/79, 89.9%, 95% CI [81.0%, 95.5%]). The prevalence of LA-MRSA-positive farms was lower than LA-MSSA-positive (36.6% of LA-SA-positive farms, 95% CI [25.5%, 48.9%] vs. 74.6%, 95% CI [62.9%, 84.2%]). In total, 190 *S*. *aureus* isolates were identified: 72 (38%) MRSA and 118 (62%) methicillin-susceptible *S*. *aureus* (MSSA), of which 174 (92%) isolates were classified to three livestock-associated lineages: CC398 (73%), CC9 (13%), and CC30/ST433 (6%). All CC398 isolates belonged to the animal clade. Four LA-MRSA clones were detected: ST433-IVa(2B) clone (n = 8, 11%), described to the best of our knowledge for the first time, and three ST398 clones (n = 64, 89%) with the most prevalent being ST398-V(5C2&5)c, followed by ST398-V(5C2), and ST398-IVa(2B).

Nasal carriage of LA-SA by pig farmers was estimated at 13.2% (38/283), CC398 carriage at 12.7% (36/283) and ST398-MRSA carriage at 3.2% (9/283), whereas by veterinarians at 21.1% (8/38), 18.4% (7/38) and 10.5% (4/38), respectively.

**Conclusions:**

The prevalence of LA-MRSA-positive pig farms in Poland has increased considerably since 2008, when the first MRSA EU baseline survey was conducted in Europe. On Polish pig farms CC398 of the animal clade predominates, this being also reflected in the prevalence of CC398 nasal carriage in farmers and veterinarians. However, finding a new ST433-IVa(2B) clone provides evidence for the continuing evolution of LA-MRSA and argues for further monitoring of *S*. *aureus* in farm animals.

## Introduction

*Staphylococcus aureus* is one of the major human opportunistic pathogens responsible for wide range of infections [1]. Of particular concern remain methicillin-resistant *S*. *aureus* (MRSA) strains causing severe infections with treatment limitations. Initially, MRSA strains had been restricted to the hospital environment (hospital-associated MRSA, HA-MRSA), but later they also emerged in the community (community-associated, CA-MRSA) [2]. During the past decade, a new subpopulation of *S*. *aureus*, so-called livestock-associated *S*. *aureus* (LA-SA), has been described. The first LA-SA strains were reported amongst pigs and pig farmers in France and the Netherlands; livestock-associated MRSA (LA-MRSA) strains were represented exclusively by clonal complex (CC) 398, whereas livestock-associated methicillin-susceptible *S*. *aureus* (LA-MSSA) were more diverse and encompassed *S*. *aureus* of both CC398 and sequence type (ST) 9 or 433 [3, 4]. In subsequent years CC398-MRSA strains were identified in pigs all over the world [5–8]. Although different MRSA lineages adapted to pigs have been reported, only two of them are recognized as the most prevalent: CC398 in Europe, and CC9 in Asia [9–11]. CC398-MRSA strains are drawing increased attention on grounds of their particular ability to colonize livestock animals, and be transmitted to persons being in contact with colonized animals and their environment [12–15]. Despite suggestions that CC398 is less virulent than other MRSA lineages, a number of studies demonstrate infections caused by CC398-MRSA in humans, especially in pig farmers and their family members [16–19] and indicate food-producing animals as a potential source of their origin.

The epidemiological situation associated with LA-SA is not well recognized in the eastern and south-eastern countries of the European Union, and the number of publications concerning the prevalence of LA-MRSA on livestock farms and in animal-food products in this region is limited. In Poland, the first ST398-MRSA strains were observed in 2008 in specimens from humans (veterinarians having contact with pigs) [20]. As our country is one of the largest pork producers, ranked fourth in the European Union in 2013, with 1.6 million tons of pig meat per year and sixth with almost 11 million pigs produced per year (Eurostat 2014, http://ec.europa.eu/eurostat/web/agriculture/data/main-tables), data from a *S*. *aureus* structured survey on Polish pig farms would expand knowledge on the LA-SA population in Europe.

The aim of the study was to determine the dissemination of LA-SA, both MRSA and MSSA, on pig farms selected throughout Poland, characterize the population structure of *S*. *aureus* identified in humans, pigs and their environment, and assess the prevalence of LA-SA carriage amongst farmers and veterinarians being in contact with pigs.

## Material and methods

### Farm selection and characteristics

The existing database of the Department of Swine Diseases, National Veterinary Research Institute was used to contact over 400 veterinarians, taking care of over 3,500 swine herds in Poland. The selection process was performed by contacting veterinary practitioners that agreed to contribute in the study. As a consequence, in the period from August 2010 to November 2012 a cross-sectional study was conducted on 123 pig farms (89 farrow-to-finish and 34 nucleus herds), located in 15 out of 16 provinces of Poland. The number of farms selected was proportional to the headage of pig per 100 ha in a particular province. The study did not include the Lower Silesia Province, where pig production is amongst the lowest in the country. The size of the farrow-to-finish herds ranged from ten to 2000 sows (median 80) and of the nucleus herds from nine to 1000 (median 54). Sixty-four (52%) farms had their own pig production, while 59 (48%) purchased gilts and/or boars from other sources. Thirty-nine (32%) farms obeyed the rules of good management applying the All-In-All-Out (AIAO) procedure and 57 (46%) farms did not. For 27 (22%) of farms no data were obtained. In the AIAO procedure the animals are kept together in groups. The groups are closely matched by age, weight, production stage and condition. The animals are moved into and out of facilities in groups. The facilities are cleaned and thoroughly disinfected between groups of animals. Most of the farms weaned their piglets between 21–49 days of life, with an average of 28 days.

### Sampling procedure

There were three sources of material: human and pig nasal swabs and environmental dust samples. From each farm, both pig nasal swabs and dust samples were collected during one-off screening (one visit at the farm) in three different sectors: farrowing unit (sows), nursery and fattening unit. Five nasal swabs from five healthy pigs and five environmental dust samples were collected from each sector. Dust samples were taken mainly from pens and ventilator ducts. Whenever possible, five pigs from each age group were selected using a convenience sampling scheme that avoided sampling more than one pig in co-mingled groups. If all three age groups were not present on the farm, pigs from the available groups were sampled.

Nasal swabs were collected at the same time, from consenting owners of the farms, individual workers taking care of animals (hereinafter all referred to as”pig farmers”) and from veterinarians supervising pig farms. The vets working on several farms were screened only once. The number of nasal swabs collected from farm personnel depended on the number of workers employed at each farm and individual agreement to be sampled given by every person.

One swab per volunteer on each farm was taken from both nares personally after swabbing training. In total, 321 human nasal swabs (283 from pig farmers and 38 from vets), 1845 pig nasal swabs and 1845 dust samples were collected. All samples were stored at 4°C and passed on directly to the laboratory. Nasal cotton swabs were transported in Amies medium (Medlab Products, Raszyn, Poland).

At the time of the project application and realization ethical approval was not required for taking nasal swabs according to local and national regulations (the project was reviewed and accepted by the Ministry of Science and Higher Education, Poland and additional approval was not recquired). All human participants signed an informed consent form. Collected data were anonymized before analysis at the National Veterinary Research Institute, Puławy.

### Bacterial isolates

Material from five pig nasal swabs, collected in each farm sector, were pooled into one sample, placed in a 100 ml Mueller-Hinton Broth (BD BBL, France) supplemented with 6.5% NaCl and incubated at 37°C for 16–20 h. The same was done with dust swabs. In total, 738 cultures were set up (369 from pigs and 369 from dust samples). One milliliter of each bacterial culture was inoculated into 9 ml Trypticase Soy Broth (BD BBL, France) supplemented with aztreonam (75 mg/l) and incubated for a further 16–20 h at 37°C. One loop-full of each positive culture, as well as nasal swabs from humans, were spread at the same time onto Chapman agar (bioMérieux, France) and chromogenic agar selective for *S*. *aureus* species (SAID, bioMérieux, France) and incubated for 24–48 h at 37°C. Grown colonies were subcultured on 5% sheep blood agar (BD BBL, France). Bacteria were stored at -80°C for further identification.

Species identification was based on phenotypic criteria including colony morphology, and both CF and coagulase tests. Additionally Microgen^TM^ Staph (Microgen Bioproducts Limited, Camberley, Surrey, UK) rapid confirmatory latex agglutination test for *S*. *aureus* was used.

All *S*. *aureus* isolates were tested for methicillin resistance by using the cefoxitin-disc diffusion method. The analysis was performed according to procedure and breakpoints proposed by EUCAST recommendations [21] with *S*. *aureus* ATCC29213 as the reference strain. Isolates were also screened by PCR for the presence of the *mecA* and the *mecC* gene as described earlier [22, 23].

### Total DNA preparation

Total DNA of the *S*. *aureus* isolates was purified using Genomic Mini DNA kit (A&A Biotechnology, Gdynia, Poland) according to the manufacturer’s instructions.

### Genetic background determination

Initially, *S*. *aureus* isolates were screened by PCR for identification of CC398 [24] and in all accessory gene regulator (*agr*) allotypes were determined [25]. Amongst CC398 isolates discrimination between human and animal clade was performed [26].

*spa* typing was performed [27] and *spa*-types were determined by using the Ridom StaphType software v.2.1.1 (Ridom GmbH) [28]. The BURP algorithm was used to assign *spa-*types into *spa*-clonal complexes (*spa*-CCs) with defined default parameters "exclude *spa-*types shorter than 5 repeats" and "cluster *spa-*types into the same group if cost distances are less than 4" [29].

Multilocus Sequence Typing (MLST) was performed on selected isolates, 36 MRSA and 62 MSSA [30]. At least one isolate per each *spa*-type detected in each source of material was analyzed. The sequence type (ST) was also determined for three non-*spa*-typeable isolates. Allele numbers and STs were assigned through the *S*. *aureus* MLST database (http://saureus.mlst.net).

Clonal complexes, CCs, were determined using the eBURSTv3 algorithm (http://saureus.mlst.net/eburst) and restricted to STs that share five or more alleles of seven loci examined with founder or subfounders, within each predicted clonal group [31]. The analysis was performed on April 29^th^, 2016.

### SCC*mec* typing

SCC*mec* types and subtypes were determined [32, 33], and those of type V were analyzed by additional strategies [34, 35]. The presence of the *czrC* gene encoding cadmium and zinc resistance was detected [36]. The SCC*mec* elements were classified according to guidelines proposed by the International Working Group on the Classification of SCC elements [37]. Direct repeat unit (*dru*) VNTR regions associated with SCC*mec* elements were determined [38] and *dru*-types were assigned through dru-typing database (http://dru-typing.org).

#### ACME and toxin genes profiling

Detection of the arginine catabolic mobile element (ACME) was performed in duplex PCR with primers AIPS.29 and AIPS.28 (locus *arc*) and AIPS.45 and AIPS.46 (locus *opp3*) [39].

The presence of the *lukS-PV* and *lukF-PV* genes encoding Panton-Valentine leukocidin (PVL) was determined [40], and the detection of the following genes coding for staphylococcal enterotoxins (SEs) was performed: *sea*, *seb*, *sec*, *sed* [41], *see*, *seg*, *seh*, *sei* [42], and *sep* [43].

#### Detection of prophage of integrase type 3 (φSa*int*3) and IEC types classification

The presence of φSa*int*3 was determined by detection of the prophage integrase type 3 (*int*3) gene [44]. The composition of *sak*, *chp*, and *scn* genes from the human-specific Immune Evasion Cluster (IEC), carried on φSa*int*3, was determined by PCR and IEC types were classified [45].

### Statistical analysis

Exact (permutation) chi-square tests were used to detect differences between frequencies of *S*. *aureus* types in farm/sample categories, and interval estimates for proportions and odds ratios were found by exact methods using SAS 9.4 (SAS Institute, 2014) [46].

Genetic diversity, on the grounds of *spa*-types, was estimated using Simpson’s index of diversity (SID) with 95% confidence intervals [47, 48].

### Definitions

Farms were defined as *S*. *aureus*-positive, when at least one *S*. *aureus* isolate was identified in a farm sample from pig farmer, pig or environmental dust. Equivalent criteria were applied for defining of LA-SA-positive farms. Isolates were classified as LA-SA when they belonged to livestock-associated CCs (LA-CCs): CC398, CC9 or CC30/ST433.

MRSA clones were defined based on ST and SCC*mec* type/subtype association [30].

## Results

### Distribution of LA-SA across the country and on farms

*S*. *aureus* was detected on 79 out of 123 (64.2%) farms from 14 provinces (Table 1). Amongst these farms LA-SA-positive dominated (71/79, 89.9%, 95% CI [81.0%, 95.5%]). On 18 farms (25.4% of LA-SA-positive farms) *S*. *aureus* was identified exclusively in samples from pig farmers. Both LA-MRSA and LA-MSSA were observed on eight farms (11.3%), while LA-MRSA and LA-MSSA alone were identified on 18 (25.4%) and 45 (63.4%) farms, respectively. LA-SA-positive farms comprised 52.9% (18/34) of nucleus herds and 59.6% (53/89) of farrow-to-finish, including 50.0% (32/64) of farms with its own production of pigs, and 66.1% (39/59) purchasing gilts and/or boars from another sources. Thirty (42.3%) LA-SA-positive farms obeyed the AIAO procedure and the same number did not. For 11 (15.5%) farms, no information was available.

**Table 1:** Geographical location and *S*. *aureus* status of pig farms (n = 123) contributed to survey in Poland, 2010–2012.

Farm ID	Geographical coordinates (longitude, latitude)	Province	*S*. *aureus* status^a^
**1**	22.17185, 52.37441	Mazovia	LA-SA-positive (MRSA and MSSA)
**2**	22.24721, 51.16994	Lublin	LA-SA-positive (MSSA)
**3**	16.68148, 52.09516	Wielkopolska	LA-SA-positive (MSSA)
**4**	17.69516, 52.72823	Kujawy-Pomerania	LA-SA-positive (MSSA)
**5**	17.35666, 52.31221	Wielkopolska	LA-SA-positive (MSSA)
**6**	16.41938, 52.64721	Wielkopolska	SA-negative
**7**	17.30123, 53.00928	Wielkopolska	LA-SA-positive (MSSA)
**8**	17.25818, 52.49172	Wielkopolska	LA-SA-positive (MSSA)
**9**	16.55444, 52.94722	Wielkopolska	SA-negative
**11**	16.42755, 52.48697	Wielkopolska	LA-SA-positive (MSSA)
**12**	17.04769, 52.84694	Wielkopolska	LA-SA-negative (MSSA)
**13**	17.89987, 53.23821	Kujawy-Pomerania	SA-negative
**14**	16.69217, 52.86952	Wielkopolska	SA-negative
**15**	17.66103, 52.71815	Kujawy-Pomerania	LA-SA-positive (MSSA)
**17**	18.20091, 52.91935	Kujawy-Pomerania	LA-SA-positive (MSSA)
**18**	19.09861, 53.00259	Kujawy-Pomerania	LA-SA-positive (MRSA and MSSA)
**19**	18.87245, 53.8043	Pomerania	LA-SA-positive (MRSA)
**20**	18.94197, 53.525	Kujawy-Pomerania	LA-SA-positive (MSSA)
**21**	18.57994, 53.85009	Pomerania	SA-negative
**22**	22.52883, 52.42419	Mazovia	LA-SA-positive (MSSA)
**23**	16.24568, 52.19203	Wielkopolska	LA-SA-positive (MSSA)
**24**	17.75802, 51.28189	Wielkopolska	LA-SA-positive (MSSA)
**25**	17.7267, 51.63854	Wielkopolska	LA-SA-negative (MSSA)
**26**	17.64507, 52.26269	Wielkopolska	LA-SA-positive (MSSA)
**27**	17.64198, 52.25681	Wielkopolska	SA-negative
**29**	16.84753, 52.83761	Wielkopolska	SA-negative
**35**	17.91904, 53.26326	Kujawy-Pomerania	SA-negative
**37**	22.43991, 52.09469	Mazovia	LA-SA-positive (MSSA)
**38**	22.67715, 52.16361	Mazovia	LA-SA-positive (MSSA)
**39**	23.33258, 52.72148	Podlasie	LA-SA-positive (MRSA)
**40**	22.81173, 52.93146	Podlasie	LA-SA-positive (MSSA)
**42**	17.3965, 52.53846	Wielkopolska	LA-SA-positive (MSSA)
**45**	16.83311, 52.58386	Wielkopolska	LA-SA-positive (MRSA and MSSA)
**51**	22.53604, 52.24104	Mazovia	LA-SA-positive (MSSA)
**52**	22.2904, 52.37013	Mazovia	SA-negative
**53**	22.39834, 52.23087	Mazovia	LA-SA-negative (MSSA)
**54**	22.10174, 52.7768	Mazovia	LA-SA-positive (MRSA and MSSA)
**55**	22.23761, 52.36721	Mazovia	SA-negative
**57**	22.78175, 51.73661	Lublin	LA-SA-positive (MSSA)
**58**	15.77598, 53.77565	West Pomerania	LA-SA-positive (MSSA)
**59**	15.86391, 53.77894	West Pomerania	LA-SA-positive (MSSA)
**70**	18.90558, 49.95658	Silesia	LA-SA-positive (MSSA)
**71**	18.94472, 49.88379	Silesia	LA-SA-positive (MRSA and MSSA)
**72**	18.09233, 54.06432	Pomerania	LA-SA-positive (MSSA)
**73**	23.61785, 50.35816	Lublin	LA-SA-positive (MRSA)
**74**	21.54848, 51.84871	Mazovia	LA-SA-positive (MSSA)
**76**	16.75025, 51.83041	Wielkopolska	LA-SA-positive (MSSA)
**77**	15.87026, 51.79996	Lubuskie	LA-SA-positive (MSSA)
**78**	22.11959, 51.4174	Lublin	LA-SA-positive (MSSA)
**79**	23.02728, 51.74109	Lublin	LA-SA-positive (MRSA)
**80**	19.70844, 51.55999	Łódź	LA-SA-positive (MSSA)
**83**	17.92129, 50.6751	Opole	SA-negative
**84**	17.81274, 50.77843	Opole	LA-SA-negative (MSSA)
**85**	17.6471, 52.2021	Wielkopolska	LA-SA-positive (MRSA)
**86**	17.6471, 52.2021	Opole	SA-negative
**87**	19.24615, 53.26038	Kujawy-Pomerania	SA-negative
**88**	19.18576, 53.39656	Kujawy-Pomerania	SA-negative
**89**	19.14848, 53.39131	Kujawy-Pomerania	SA-negative
**90**	17.70678, 52.26143	Wielkopolska	SA-negative
**91**	17.63243, 52.17326	Wielkopolska	SA-negative
**92**	17.6227, 52.21698	Wielkopolska	SA-negative
**93**	20.28059, 50.31664	Małopolska	SA-negative
**94**	18.30356, 54.35745	Pomerania	SA-negative
**95**	20.30899, 50.29191	Małopolska	LA-SA-positive (MRSA and MSSA)
**96**	20.32007, 50.31121	Małopolska	SA-negative
**98**	23.53364, 51.54219	Lublin	SA-negative
**99**	15.40782, 52.52347	Lubuskie	LA-SA-positive (MRSA)
**100**	19.60076, 52.13416	Łódź	LA-SA-positive (MRSA)
**101**	19.87134, 52.18087	Łódź	LA-SA-positive (MRSA and MSSA)
**102**	17.1, 52.8	Wielkopolska	LA-SA-positive (MRSA)
**103**	22.32872, 51.20957	Lublin	LA-SA-positive (MSSA)
**104**	17.50705, 52.73566	Kujawy-Pomerania	LA-SA-positive (MRSA)
**105**	18.19092, 52.46879	Wielkopolska	LA-SA-negative (MSSA)
**106**	18.08727, 51.8433	Wielkopolska	LA-SA-positive (MSSA)
**107**	17.62275, 50.93008	Opole	LA-SA-positive (MRSA)
**108**	22.91782, 52.26941	Mazovia	LA-SA-positive (MRSA)
**109**	21.87051, 50.86556	Lublin	SA-negative
**112**	22.95421, 53.42958	Podlasie	LA-SA-positive (MSSA)
**113**	23.01446, 53.3929	Podlasie	LA-SA-positive (MRSA)
**114**	18.22168, 52.4764	Wielkopolska	SA-negative
**115**	19.32846, 51.95981	Łódź	LA-SA-positive (MRSA and MSSA)
**116**	22.89344, 53.44369	Podlasie	LA-SA-positive (MSSA)
**118**	23.31642, 53.58639	Podlasie	LA-SA-positive (MRSA)
**120**	22.99412, 53.37544	Podlasie	LA-SA-positive (MSSA)
**121**	22.99704, 53.3739	Podlasie	LA-SA-positive (MSSA)
**122**	19.71647, 50.12223	Małopolska	SA-negative
**123**	20.44778, 50.35685	Świętokrzyskie	SA-negative
**124**	22.81723, 51.73649	Lublin	SA-negative
**125**	19.80491, 52.23452	Łódź	SA-negative
**126**	20.29643, 50.28102	Małopolska	LA-SA-positive (MSSA)
**127**	20.29385, 50.28766	Małopolska	LA-SA-positive (MRSA)
**128**	19.89823, 51.61135	Łódź	LA-SA-negative (MSSA)
**129**	22.94305, 51.81137	Lublin	SA-negative
**130**	23.05343, 51.82325	Lublin	SA-negative
**131**	22.81508, 51.7329	Lublin	LA-SA-positive (MRSA)
**133**	16.5138, 52.03222	Wielkopolska	SA-negative
**134**	17.71056, 52.25681	Wielkopolska	LA-SA-positive (MRSA)
**136**	18.21756, 52.47543	Wielkopolska	SA-negative
**137**	19.87778, 52.18056	Łódź	SA-negative
**138**	22.81293, 51.72973	Lublin	SA-negative
**139**	20.43697, 50.34524	Świętokrzyskie	SA-negative
**141**	18.76396, 54.00332	Pomerania	LA-SA-positive (MSSA)
**142**	18.38905, 54.74682	Pomerania	SA-negative
**143**	23.16882, 51.94683	Lublin	LA-SA-positive (MSSA)
**144**	21.80011, 51.75259	Mazovia	LA-SA-positive (MSSA)
**145**	20.44898, 53.45228	Warmia-Masuria	LA-SA-positive (MSSA)
**146**	23.26563, 52.05259	Lublin	LA-SA-positive (MRSA)
**147**	21.75601, 51.91907	Mazovia	SA-negative
**148**	18.4136, 50.43553	Silesia	LA-SA-negative (MSSA)
**149**	19.16272, 53.4733	Warmia-Masuria	LA-SA-positive (MSSA)
**150**	18.74198, 52.70806	Kujawy-Pomerania	SA-negative
**151**	21.80356, 51.93124	Mazovia	SA-negative
**152**	20.03078, 53.65724	Warmia-Masuria	LA-SA-positive (MSSA)
**154**	18.10426, 53.41812	Kujawy-Pomerania	SA-negative
**155**	18.9109, 52.74865	Kujawy-Pomerania	LA-SA-positive (MSSA)
**157**	23.02871, 50.32058	Lublin	LA-SA-positive (MSSA)
**158**	18.70954, 53.08536	Kujawy-Pomerania	LA-SA-positive (MRSA)
**159**	21.29794, 49.87913	Podkarpacie	LA-SA-negative (MSSA)
**160**	17.58224, 52.23452	Wielkopolska	SA-negative
**161**	17.65757, 52.09642	Wielkopolska	LA-SA-positive (MSSA)
**163**	21.33562, 50.11023	Podkarpacie	SA-negative
**164**	18.61368, 51.9739	Wielkopolska	SA-negative
**165**	21.66366, 51.63394	Lublin	SA-negative

^a^LA-SA-positive or -negative—livestock-associated *S*. *aureus*-positive or -negative farm, respectively; SA-negative*–S*. *aureus*-negative farm. Phenotypes of resistance to methicillin of *S*. *aureus* isolates identified on *S*. *aureus*-positives farms are in brackets.

LA-MRSA isolates were detected on 26 farms (21.1% of all) from 11 provinces, while LA-MSSA on 53 farms (43.1% of all) from 12 provinces. Amongst all LA-SA-positive farms, the prevalence of LA-MRSA-positive farms was lower than LA-MSSA-positive ones (36.6%, 95% CI [25.5%, 48.9%] vs. 74.6%, 95% CI [62.9%, 84.2%]). LA-MRSA-positive farms comprised 26.5% (9/34) of nucleus and 19.1% (17/89) farrow-to-finish herds, with isolates from pig farmers, pigs and dust detected on five (14.7%), four (11.8%) and seven (20.6%) nucleus, and three (3.4%), 11 (12.4%) and 12 (13.5%) farrow-to-finish farms, respectively. Regarding LA-MSSA-positive farms, they comprised 35.3% (12/34) of nucleus and 46.1% (41/89) farrow-to-finish herds, with isolates from pig farmers, pigs and dust detected on seven (20.6%), six (17.6%) and six (17.6%) nucleus, and 20 (22.5%), 23 (25.8%) and nine (10.1%) farrow-to-finish farms, respectively.

No differences were demonstrated in distribution of LA-SA-positive farms throughout the country but, due to modest sample size their existence cannot be excluded (wide confidence intervals for odds ratios, not shown). Regarding LA-SA-negative farms, which encompassed 35.8% (44/123) of all farms studied, we did not notice any differences concerning geographic location, type of farm, size of herd or application of AIAO procedure, compared with LA-SA-positive ones.

### Molecular analysis

**Fig 1:**
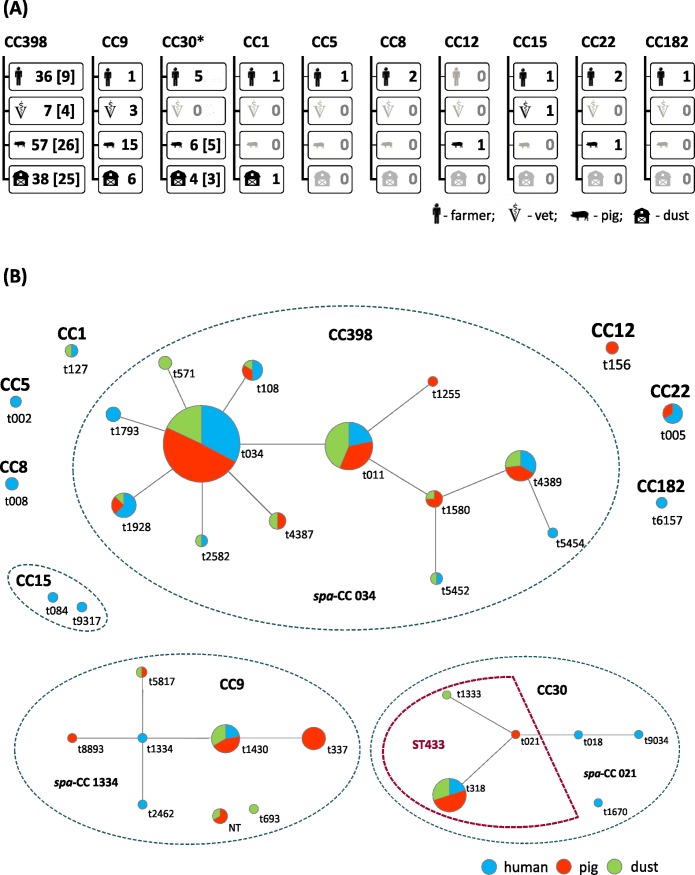
*S*. *aureus* genetic lineages and *spa*-clonal complexes (*spa*-CCs) determined on Polish pig farms. **(A)** Distribution and the total number of *S*. *aureus* isolates from humans (farmers and veterinarians), pigs and environmental dust amongst MLST clonal complexes (CCs). The number of MRSA isolates is in brackets. *One isolate from pig farmer and all isolates from pigs and dust belonged to ST433. **(B)** Population structure based on *spa*-CCs BURP analysis; MLST CCs as well as ST433 within CC30 are indicated.

In total, 190 isolates were identified as *S*. *aureus*, including MRSA (n = 72, 37.9%) and MSSA (n = 118, 62.1%). The isolates were classified into 10 genetic lineages: CC398 (72.6% of all isolates), CC9 (13.2%), CC30 (7.9%), CC22 (1.6%), CC1, CC8, CC15 (1.1% each), CC5, CC12, and CC182 (0.5% each). Four of these lineages, CC5, CC8, CC15, and CC182, were detected exclusively amongst isolates from humans (Fig 1A). Thirty-four *spa*-types were identified (Fig 1B), 7 and 34 amongst MRSA and MSSA, respectively.

None of the *S*. *aureus* isolates carried *lukS/lukF*-PV genes and ACME element. MRSA isolates carried *mec*A gene and SCC*mec* elements classified to type IV or V. The *int*3 gene was identified exclusively in ten MSSA isolates, including one ST398-MSSA from a pig farmer. Based on the presence of *sea*, *sep*, *scn*, *chp*, and *sak* genes five IEC types (A, B, D, E and F) have been determined, with the predominant type B (60.0%). IECs were more prevalent in non-LA-MSSA than LA-MSSA isolates (p = 0.0063), and in human than non-human isolates (p = 0.0011). The molecular characteristics of MRSA and MSSA isolates from *S*. *aureus*-positive pig farms are shown in Tables 2 and 3, respectively.

**Table 2:** Molecular characteristics of methicillin-resistant *S*. *aureus* isolates (n = 72) from 26 MRSA-positive pig farms in Poland.

CC (n)	*agr*	ST	*spa-*type	SCC*mec*-type	*dru*-type	SEs genes	Humans^a^ n = 13(4)	Pigs n = 31	Dust n = 28
**CC398 (64)**	I	ST398	t011	IVa(2B)	dt10q	-	-	-	1
				V(5C2&5)c	dt11a	-	6 (3)	10	10
					dt11cp	-	-	1	1
				V(5C2&5)c_var1_	dt8f	-	-	-	1
			t034	V(5C2)	dt11p	-	-	5	2
				V(5C2&5)c	dt11a	-	3	3	2
					dt11af	-	-	2	2
					dt9s	-	1	-	2
					dt11ap	-	-	1	-
					dt5e	-	1 (1)	-	-
				V(5C2&5)c_var2_	dt11co	-	-	1	-
			t108	V(5C2&5)c	dt11a	-	2	2	1
			t2582	V(5C2&5)c	dt9s	-	-	-	1
			t4389	V(5C2&5)c	dt11a	-	-	1	1
			t5452	V(5C2&5)c	dt11a	-	-	-	1
**CC30 (8)**	III	ST433 (SLV ST30)	t318	IVa(2B)	dt10a	*seg*, *sei*	-	5	3

^a^Total number of isolates from humans, number of isolates from veterinarians are indicated in brackets. n—number of isolates; CC–Clonal Complex; ST–Sequence Type; SLV—single locus variant.

**Table 3:** Molecular characteristics of methicillin-susceptible *S*. *aureus* isolates (n = 118) from 61 MSSA-positive pig farms in Poland.

CC (n)	*agr*	ST	*spa-*type	SEs genes	*int* φ3	*sak*	*chp*	*scn*	IEC type^a^	Humans^b^ n = 48 (7)	Pigs n = 49	Dust n = 21
**CC398 (74)**	I	ST398	t011	-	-	-	-	-	-	-	-	1
			t034	-	-	-	-	-	-	14 (1)	18	3
			t108	-	-	-	-	-	-	1	-	-
			t571	-	-	-	-	-	-	-	-	2
			t1580	-	-	-	-	-	-	-	3	1
			t1793	-	-	-	-	-	-	3	-	-
			t1928	-	-	-	-	-	-	4 (1)	2	1
					+	+	+	+	B	1	-	-
			t2582	-	-	-	-	-	-	1	-	-
			t4387	-	-	-	-	-	-	-	2	2
			t4389	-	-	-	-	-	-	3 (1)	5	2
			t5452	-	-	-	-	-	-	1	-	-
		ST2769^**c**^ (SLV ST398)	t1255	-	-	-	-	-	-	-	1	-
		ST2850 (SLV ST398)	t5454	-	-	-	-	-	-	1	-	-
		ST3168^**c**^ (SLV ST398)	t4389	-	-	-	-	-	-	1	-	1
**CC9 (25)**	II	ST9	t337	*seg*, *sei*	-	-	-	-	-	-	7	-
			t693	*seg*, *sei*	-	-	-	-	-	-	-	1
			t1334	*seg*, *sei*	-	-	-	-	-	1 (1)	-	-
			t1430	*seg*, *sei*	-	-	-	-	-	2 (1)	4	3
			t2462	*seg*, *sei*	-	-	-	-	-	1 (1)	-	-
			t8893	*seg*, *sei*	-	-	-	-	-	-	1	-
			NT	*seg*, *sei*	-	-	-	-	-	-	2	1
		ST2423 (SLV ST9)	t5817	*seg*, *sei*	-	-	-	-	-	-	1	1
**CC30 (7)**	III	ST433 (SLV ST30)	t021	*seg*, *sei*	-	-	-	-	-	-	1	-
			t318	*seg*, *sei*	-	-	-	-	-	1	-	-
			t1333	*seg*, *sei*	-	-	-	-	-	-	-	1
		ST30	t018	*sea*, *seg*, *sei*	+	+	+	+	A	1	-	-
			t9034	*seg*, *sei*	-	-	-	-	-	1	-	-
		ST2612^**c**^ (SLV ST30)	t318	*seg*, *sei*	+	+	-	+	E	1	-	-
		ST1708 (DLV ST30)	t1670	*seh*, *seg*, *sei*	-	-	-	-	-	1	-	-
**CC1 (2)**	III	ST1	t127	*sea*, *seh*, *sek*, *seq*	+	+	-	+	D	1	-	-
				*seh*	-	-	-	-	-	-	-	1
**CC5 (1)**	II	ST5	t002	*seg*, *sei*	+	+	+	+	B	1	-	-
**CC8 (2)**	I	ST3167^**c**^ (SLV ST8)	t008	-	-	-	-	-	-	2	-	-
**CC12 (1)**	II	ST12	t156	*sec*, *sep*	+	+	+	+	F	-	1	-
**CC15 (2)**	II	ST15	t084	-	-	-	-	-	-	1	-	-
			t9317	-	-	-	-	-	-	1 (1)	-	-
**CC22 (3)**	I	ST22	t005	*seg*, *sei*	+	+	+	+	B	2	-	-
		ST2950^**c**^ (SLV ST22)	t005	*seg*, *sei*	+	+	+	+	B	-	1	-
**CC182 (1)**	I	ST182	t6157	*seh*, *seg*, *sei*	+	+	+	+	B	1	-	-

^a^Immune Evasion Cluster types in accordance with the classification proposed by van Wamel et al. [45].

^b^Total number of isolates from humans; number of isolates from veterinarians are indicated in brackets.

^c^Novel ST due to identification of a new allele. n—number of isolates; CC–Clonal Complex; ST–Sequence Type; SLV and DLV—single locus variant and double locus variant, respectively. NT—non-typeable isolates, collected on one pig farm.

### Characteristics of *S*. *aureus* isolates from LA-CCs

Three livestock-associated genetic lineages, CC398, CC9, and CC30/ST433, comprised 91.6% (174/190) of *S*. *aureus* isolates studied. LA-MRSA isolates were found in two of them, CC398 and CC30/ST433. CC398 isolates, both MRSA and MSSA, as well as MSSA isolates of CC9 and CC30/ST433 were identified in all types of samples, while ST433-MRSA only in non-human samples (from pigs and dust). CC398-MRSA were more frequently observed in dust samples than in samples from pig farmers (p = 0.006).

Twenty-three *spa*-types were identified amongst LA-MSSA isolates of which seven, t011, t034, t108, t318, t2582, t4389 and t5452, were shared with LA-MRSA. The LA-MSSA population was more diverse than LA-MRSA in terms of *spa*-types (SID = 0.843, 95% CI [0.785, 0.906] vs. SID = 0.697, 95% CI [0.634, 0.761]).

**CC398**, the most prevalent CC (n = 138), was detected on 59 (48.0% of all) farms from 13 provinces, with MRSA found on 24 (19.5%) farms from 11 provinces and MSSA on 40 (32.5%) farms from 11 provinces. All CC398 isolates were assigned to the animal clade. Thirteen different *spa*-types were identified and clustered by BURP analysis into one clonal complex *spa*-CC 034. Over 75% isolates were classified to three predominant *spa*-types: t034 (43.5%), t011 (22.5%), and t4389 (10.1%). All six *spa*-types determined amongst CC398-MRSA were also found amongst CC398-MSSA isolates, however amongst MRSA dominated t011 (46.9%) and t034 (39.1%), whereas amongst MSSA—t034 (47.3%), t4389 (16.2%), and t1928 (10.8%). With one exception, SCC*mec* elements of CC398 belonged to type V (n = 63, 87.5% of MRSA). Fifty-four of the SCC*mec* V (85.7%) were classified as type V(5C2&5)c. All of them carried *czrC*, *ccrC2* and *ccrC8* but not *orf33* and *orf35* genes. They were characterized by eight *dru*-types, with the most prevalent dt11a (n = 42). Seven of the SCC*mec* V elements (11.1%) were classified as type V(5C2). All of them were characterized by the presence of *ccrC*, *orf33* and *orf35* genes and were of *dru*-type dt11p, exclusively identified in this type V. In none of them was the *czrC* gene detected.

On 10 farms ST398 isolates were identified in both pig farmers and non-human reservoirs. The isolates from the same farm shared their characteristics (*spa*-type, SCC*mec*-type and *dru*-type in case of MRSA or *spa*-type in case of MSSA).

**CC9**, the second largest CC, was detected on 13 (10.6%) farms from seven provinces and embraced only MSSA isolates. Seven *spa*-types were identified, with the predominant types: t1430 (36.0%) and, observed exclusively in pigs, t337 (28.0%). All *spa*-types but one were grouped into one *spa*-CC 1334.

**CC30/ST433** genetic lineage was distinguished within CC30, the third numerous CC in this study. MRSA and MSSA isolates of CC30/ST433, were detected on two and three farms, respectively, each from a different province. Isolates were assigned to three *spa*-types clustered into one *spa*-CC 021, with the most prevalent being t318. All MRSA isolates belonged to a new clone ST433-IVa(2B).

### LA-SA carriage in the human population

Amongst *S*. *aureus* detected in humans, 50 isolates were from pig farmers and 11 from vets (Fig 1A). The majority of *S*. *aureus* isolates (n = 36, 72.0%) found among pig farmers belonged to genetic lineage CC398; the remaining represented CC1, CC5, CC8, CC9, CC15, CC22, CC30, and CC182. Isolates from vets were classified to three CCs: CC398, CC9, and CC15. All MRSA isolates (n = 13) identified in the human population belonged to ST398. Nasal carriage of LA-SA by pig farmers was estimated at 13.2% (38/283), CC398 carriage at 12.7% (36/283) and ST398-MRSA carriage at 3.2% (9/283), whereas by veterinarians at 21.1% (8/38), 18.4% (7/38) and 10.5% (4/38), respectively. Amongst pig farmers, CC398-MSSA carriage (9.5% (27/283); 95%CI [6.4%, 13.6%]) was higher than CC398-MRSA carriage (3.2%; 95%CI [1.5%, 6.0%]).

## Discussion

The epidemiology of *S*. *aureus* in Western and Central Europe has changed significantly in the past decade due to the emergence of genetic lineages adapted to livestock animals, especially to pigs. Here we present the results of the first structured survey conducted on pig farms in Poland, which partially fill the gap concerning the prevalence and molecular characteristics of livestock-associated *S*. *aureus*, both MRSA and MSSA, in the central and eastern EU countries.

In 2008, when the first MRSA EU baseline survey on dust samples from pig holdings was conducted in Europe, Poland was classified amongst countries with the lowest occurrence of MRSA-positive farms [10]. Since that time, as our study shows, the prevalence of LA-MRSA-positive farms has increased considerably, from 2.1% to 20.6% in breeding (nucleus) holdings (95% CI for Odds Ratio [2.6, 77]), and from 3.4% to 13.5% in production (farrow-to-finish) holdings (95% CI for OR [1.48, 14.9]). However, it still remains lower than those observed in 2008 on pig farms in Belgium, Germany, or Spain [10]. One valid explanation of the observed higher percentage of LA-MRSA-positive farms may be the increase in imports of piglets, over the past years to Poland, from countries like Denmark, Germany and the Netherlands [49], where the rates of LA-MRSA colonized pigs are high [14, 50]. The lower prevalence of LA-MRSA-positive than LA-MSSA-positive farms, observed in our study, may arise from the fact that one-fourth of LA-SA-positive farms have been classified based on *S*. *aureus* isolates detected exclusively in samples from pig farmers, where the occurrence of LA-MSSA is higher than LA-MRSA.

The nasal carriage of ST398-MRSA by farmers exposed to pigs in this study was low (3.2%) compared with rates in similar populations from other countries, ranging from about 20% in Canada and USA [5, 7] to over 80% in Belgium, Denmark and Germany [12, 51]. Concurrently, CC398-MRSA carriage amongst Polish pig farmers was three times lower than CC398-MSSA. The reason for this remains unclear, however, it was earlier observed in Germany that the frequency of transfer events of CC398-MSSA amongst non-exposed to pigs people living on pig farms was twice as high as for CC398-MRSA [12]. Our results may support these findings and/or may suggest that within the CC398 lineage MSSA strains are better adopted to humans than MRSA. However, since our findings are contrary to results described by Denis et al., who showed considerably higher nasal carriage of ST398-MRSA than ST398-MSSA in pig farmers from Belgium [52], only further studies could clarify this issue.

Regarding the prevalence of ST398-MRSA amongst Polish veterinarians (10.5%), it seems to be on a moderate level, comparable with that reported in Belgian livestock veterinarians (7.5%), but lower than observed in veterinarians exposed to pigs in Germany, estimated at 45% [12, 53].

Three CCs, CC398, CC9, and CC30/ST433, have been identified as the most frequent, detected on over half of the pig farms studied. All these CCs are presently recognized as the main pig-associated ones worldwide. In Western and Central Europe the predominant CC398 comprise both MRSA and MSSA, while the other two CCs are generally represented by MSSA strains [54–56].

LA-SA isolates analyzed in the current study shared features typical for pig-associated strains, like the absence of toxin genes such as exfoliative toxins, toxic-shock syndrome 1 toxin as well as Panton-Valentine leukocidin genes [57, 58]. With the one exception, CC398-MSSA-t1928 from human, they carried neither φSa*int*3 prophage nor the IEC genes, which is also characteristic for isolates adapted to animals [59–61]. All CC398 isolates analyzed belonged to the animal clade and the majority of them represented three classical, in Western and Central Europe, pig-borne *spa*-types, t011, t034 and t108 [14, 62–66]. However, the distribution of major *spa*-types varied between MRSA and MSSA. While in the MRSA population two predominant *spa*-types, t011 and t034, occurred at similar levels, amongst MSSA exclusively t034 dominated. This observation is in contrast to other reports, where the *spa*-type distribution of CC398-MSSA mirrored the most frequently detected *spa*-types for CC398-MRSA, like t011 and t034 in Belgium [56] or t034 in Denmark [54, 67].

Even though almost 90% of LA-MRSA isolates characterized in this study were assigned to CC398, the remaining MRSA, from CC30/ST433 genetic background should deserve particular attention. These MRSA isolates belonged to clone ST433-t318-IVa, that to the best of our knowledge is described here for the first time and may constitute a new LA-MRSA clone of pig origin. The isolates of the emerged clone were identified in non-human samples on two pig farms located in two, non-bordering provinces; one in the northern-eastern (Podlaskie) and second in the southern (Małopolskie) part of Poland. There were no relationships between the two farms and both purchased gilts and/or boars from other sources. Whereas CC30/ST433 is a well established LA-MSSA genetic lineage in Europe for more than 20 years [3, 54], MRSA of this lineage had not been observed till 2009. The first and the only one, until now, clone ST433-MRSA, characterized by *spa*-type t1333 and SCC*mec*V with *czr*C gene, conferring cadmium and zinc resistance, was isolated from pigs in Denmark [68], where the ST433-t1333 genotype had been earlier identified as the second most common MSSA genotype amongst pigs [54]. During our study only one ST433-MSSA isolate of *spa*-type t318 was found, however, this *spa*-type was the most frequent amongst MSSA from pig nasal swabs collected at a slaughterhouse in South-Western Poland [69]. We do not have evidence that the new clone emerged in Poland, nevertheless we cannot exclude that it arose *de novo* by the acquisition of the SCC*mec* IVa element by *S*. *aureus* of the genetic background disseminated in our country.

Identification of a new MRSA-ST433-IVa clone provides further proof for the spread of SCC*mec* elements in pig-associated *S*. *aureus* genetic lineage. Additional characterization of CC30/ST433 strains, both MRSA and MSSA, from different geographical regions may be crucial for better understanding the evolution of this lineage and its impact on global epidemiology.

We are aware of some of the limitations of this work. First, the study was extended in time for over two years and some of the farms could have changed their status from MRSA-positive to MRSA-negative and vice versa during the study period. Second, the prevalence of *S*. *aureus* isolates from pigs and environmental dust could be underestimated due to the sampling method and the pooling procedure used in the study. Nevertheless, the study allowed to establish a reference point for epidemiological investigations to be held on livestock animals, especially pigs, in the future.

In conclusion, *S*. *aureus* from pig farms in Poland belong to well established in Western and Central Europe LA-SA genetic lineages, CC398, CC9 and CC30/ST433. CC398 of the animal clade predominates on Polish pig farms, which is also reflected in the prevalence of CC398 nasal carriage in both farmers and veterinarians, having professional contacts with pigs. However, finding a new CC30/ST433-IVa(2B) clone and increasing the rate of CC398-MRSA during the last five years provides evidence for the continuing evolution and expansion of LA-MRSA in Poland. Further monitoring of *S*. *aureus* in farm animals is strongly required due to possible impact on public health.
